# BMC Ecology reviewer acknowledgement 2014

**DOI:** 10.1186/s12898-015-0035-y

**Published:** 2015-02-13

**Authors:** Catherine J Potenski

**Affiliations:** BioMed Central, 233 Spring Street, New York, NY 10013-1578 USA

**Raf Aerts**

Belgium

**Michael Anderson**

USA

**Natasha Arora**

Switzerland

**Ignacio Barberis**

Argentina

**Luke Barrett**

Australia

**Felipe Bastida**

Spain

**Jasminca Behrmann-Godel**

Germany

**Klaus Birkhofer**

Sweden

**F. Guillaume Blanchet**

Finland

**Xavier Bonnet**

France

**Henry Brink**

Tanzania

**Aurélie Coulon**

France

**Heide-Marie Daniel**

Belgium

**Eduardo De La Peña**

Belgium

**Ellen Decaestecker**

Belgium

**Sebastien Devillard**

France

**Vincent Dubut**

France

**Simon Ducatez**

Australia

**Luciana Elizalde**

Argentina

**Annette Engel**

USA

**Karl Evans**

UK

**Zhi-Wei Fan**

China

**Nicole Fogarty**

USA

**Sean Fogarty**

USA

**Wayne Fuller**

Cyprus

**Gabriel Garcia-Peña**

France

**Sean Giery**

USA

**Markus Gusset**

Switzerland

**Gabriel Hamer**

USA

**Graeme Hays**

Australia

**George Heimpel**

USA

**Roslyn Henry**

UK

**Olivier Honnay**

Belgium

**Katrine Hoset**

Finland

**Jane Hughes**

Australia

**Bas Ibelings**

Switzerland

**David Jenkins**

USA

**Guangze Jin**

China

**Maaria Kankare**

Finland

**Peter Kappeler**

Germany

**Yuta Kawarasaki**

USA

**Etienne Klein**

France

**Casper Kraan**

New Zealand

**Siegy Krauss**

Australia

**Jacco Kromkamp**

Netherlands

**Carolyn Kurle**

USA

**Bradley Lamphere**

USA

**Kevin Langergraber**

Germany

**Jean-François Le**

Galliard France

**Delphine Legrand**

France

**Junmin Li**

China

**Zhenqing Li**

China

**Marjorie Matocq**

USA

**Dominique Mazzi**

Switzerland

**Michael Mckinney**

USA

**Christoph Meier**

Switzerland

**Ben Moore**

Australia

**Xoaquin Moreira**

Spain

**Juan Moreno**

Spain

**Atsushi Ohwaki**

Japan

**Craig Packer**

USA

**Timothy Page**

Australia

**Vlada Peneva**

Bulgaria

**Charles Perrier**

Canada

**Thomas Pike**

UK

**Martin Pullan**

UK

**Thomas Quinn**

USA

**Matthew Reudink**

Canada

**Raphael Ritson-Williams**

USA

**Jordi Salmona**

Portugal

**Jose Sampaio**

Portugal

**Heiko Schmaljohann**

Germany

**Melanie Seiler**

Germany

**Aaron Shafer**

Sweden

**Shadi Shokralla**

Canada

**Jenni Stockan**

UK

**Jennifer Talbot**

USA

**Linda Tonk**

Australia

**Bettina Wachter**

Germany

**Jennifer Williams**

Canada

**Miguel A. Zavala**

Spain

**Jizhong Zhou**

USA

